# A Highly Porous Nonwoven Thermoplastic Polyurethane/Polypropylene-Based Triboelectric Nanogenerator for Energy Harvesting by Human Walking

**DOI:** 10.3390/polym12051044

**Published:** 2020-05-02

**Authors:** Hyun Ju Oh, Jong Hyuk Bae, Young Ki Park, Jinkyu Song, Do Kun Kim, Woosung Lee, Minhee Kim, Ki Joon Heo, Yoonjin Kim, Seong Hun Kim, Byeong Jin Yeang, Seung Ju Lim

**Affiliations:** 1 Advanced Textile R&D Department, Korea Institute of Industrial Technology (KITECH), Ansan 15588, Korea; hjoh33@kitech.re.kr (H.J.O.); baejh@kitech.re.kr (J.H.B.); dogun419@kitech.re.kr (D.K.K.); wslee@kitech.re.kr (W.L.); chatelier@kitech.re.kr (M.K.); heokj@kitech.re.kr (K.J.H.); pooh8476@kitech.re.kr (Y.K.); 2 Department of Organic and Nano Engineering, Hanyang University, Seoul 04763, Korea; 3 Test-Bed Research Center, Korea Dyeing & Finishing Technology Institute (DYETEC), Daegu 41706, Korea; parkyk@dyetec.or.kr; 4 Division of Nano-Convergence Material Development, National Nano Fab Center (NNFC), Daejeon 34141, Korea; jksong@nnfc.re.kr; 5 Department of Advanced Materials Engineering for Information & Electronics, Kyung Hee University, Yongin 17104, Korea

**Keywords:** polypropylene, thermoplastic polyurethane, nanofiber, porous nonwoven, mass production, spacer-free, breathable, triboelectric nanogenerator

## Abstract

A highly porous nonwoven thermoplastic polyurethane (TPU)/Polypropylene (PP) triboelectric nanogenerator (N-TENG) was developed. To fabricate the triboelectric layers, the TPU nanofiber was directly electrospun onto the nonwoven PP at different basis weights (15, 30, and 50 g/m2). The surface morphologies and porosities of the nonwoven PP and TPU nanofiber mats were characterized by field-emission scanning electron microscopy and porosimetry. The triboelectric performance of the nonwoven TPU/PP based TENG was found to improve with an increase in the basis weight of nonwoven PP. The maximum output voltage and current of the TPU/PP N-TENG with 50% PP basis weight reached 110.18 ± 6.06 V and 7.28 ± 0.67 µA, respectively, due to high air volume of nonwoven without spacers. In order to demonstrate its practical application as a generator, a TPU/PP N-TENG-attached insole for footwear was fabricated. The N-TENG was used as a power source to turn on 57 light-emitting diodes through human-walking, without any charging system. Thus, owing to its excellent energy-conversion performance, simple fabrication process, and low cost, the breathable and wearable nonwoven fiber-based TENG is suitable for large-scale production, to be used in wearable devices.

## 1. Introduction

Triboelectric nanogenerators (TENG) have attracted increased attention as effective self-powered devices for converting the ambient wasted mechanical energy into useful electric energy because of their high electrical output performance, high efficiency of energy conversion, simple structure, lightweight, and ease of fabrication [1,2,3,4,5,6,7,8,9,10]. According to the working principle of a TENG, dielectric polymers in the triboelectric series that show tribo-polarity under external mechanical force can generate energy by the coupled effect of contact electrification and electrostatic induction between two materials [11,12]. The electrical performance of a TENG not only depends on the characteristics of its constituent materials, such as the contacting materials, but also on the fabrication technique, surface modification, and structural design. It can be designed to harvest energy in four working modes: vertical contact-separation mode [13], in-plane sliding mode [14], single-electrode mode [15,16], and freestanding triboelectric-layer mode. However, the human-oriented wearable devices should be attached onto or carried by a human body for achieving optimal power-conversion efficiency. Until now, most of these devices have been fabricated in rigid planar forms with low permeability and poor flexibility, and these features restrict the normal human motion.

Fiber-based wearable TENG are one of the most challenging devices to develop for wearable electronics application. Moving away from traditional devices which use rechargeable batteries to a system of self-powering requires extensive design iterations. However, a fiber-based TENG has a distinct advantage: It has the inherent structural characteristics of fibers such as wearability, stretchability, breathability, and flexibility. Therefore, it can be a highly attractive platform for energy-harvesting [4,7,17,18,19]. Furthermore, TENGs have been fabricated based on various structural designs: core–shell single fiber structure [20], woven and knit structure [21,22], and membranous structure [23]. Considering mass production, the membranous structure based on nonwoven or nanofiber mat can be a promising structure for fabricating wearable TENGs. Several studies have shown that nanofiber-based TENGs exhibit a high electrical output and outstanding breathability, owing to their high specific surface area, inherently rough structure, and porosity [24,25,26,27,28,29,30]. Huang et al. presented a simple fabrication method of a book-shaped TENG, using polyvinylidene fluoride (PVDF) and poly(3-hydroxybutyrate-co-3-hydroxyvalerate) (PHBV) nanofibers, combined with graphene oxide (GO) sheet, and also demonstrated the electrical performance of this TENG device [24]. Mi et al. investigated the electrical performance of a TENG composed of highly porous cellulose nanofibril (CNF)/polyethylenimine (PEI) aerogel and PVDF nanofibers; it exhibited excellent triboelectric output performance, and the output voltage and current were enhanced by 18.3 and 97.6 times, respectively, by increasing the number of nanofiber layers [26]. Cheon et al. developed high-performance TENGs, using a PVDF-silver nanowire (AgNW) and nylon nanofiber triboelectric layers, and used the TENGs to create a self-powering commercial liquid crystal display [27]. Although fiber-based TENGs have been extensively studied, further studies are required for the development of a general fabrication method and material selection from the point of view of their mass production.

Melt blowing is a common method for producing nonwoven fabrics with a highly porous structure and high specific area. This process has the advantage of high production rate, low cost, and eco-friendliness (it is solvent-free), and it uses thermoplastics such as polypropylene (PP) [31], polyethylene [32], polyethylene terephthalate [33], and poly(lactic acid) [34]. Among the polymers, melt-blown PP is the most extensively used one because of its low cost, easy processability, good mechanical properties, and chemical inertness [35]. Moreover, nonwoven PP has a randomly stacked structure with high porosity and permeability. In the triboelectric series, it can serve as an electron-accepting material, and its nonwoven structure has an irregular pore distribution, which can lead to internal deformation even without a spacer.

In this study, we developed a highly porous nonwoven fiber-based nanogenerator without a spacer for energy harvesting. Nonwoven PPs with various basis weights (15, 30, and 50 g/m^2^) were manufactured by a melt-blowing process. Then, an electrospun thermoplastic polyurethane (TPU) nanofiber mat was directly fabricated on the nonwoven PP without any spacer; the porous structure served as a spacer. The morphological and structural characteristics of the nano- and microfiber-based nonwoven TPU/PP layers were investigated by field-emission scanning electron microscopy (FE-SEM), 3D digital microscope, and porosimetry. The triboelectric performance of the TPU/PP N-TENG fabricated with different basis weights of PP was also evaluated.

## 2. Materials and Methods

### 2.1. Materials

PP (HP561X, melt-blown grade, Polymirae, Korea) with a density of 0.9 g/cm^3^ and melt flow rate of 800 g/10 min was subjected to a melt-blowing process, to produce nonwoven fabric. TPU (Elastollan 1195A10) purchased from BASF Corp., (Germany) was used to fabricate the nanofiber web. A mixture of N,N-dimethylformamide (DMF) and tetrahydrofuran (THF) purchased from Thermo Fisher Scientific (USA) was used as the solvent for preparing the TPU solution.

### 2.2. Fabrication of Nonwoven Triboelectric Layers

In order to fabricate the microfiber-based nonwoven PP fabric, the melt-blowing process was performed with a pilot-scale melt-blow line (KIT MB 2005, Hills Inc., West Melbourne, FL, USA). PP resin was melted at 250 ℃ in the extruder, and the polymer melt was extruded through a spinneret, with an air gap of 0.4 mm. The nozzle to collector distance was 300 mm. The blowing-air temperature to obtain the nonwoven fabric was set to 260 ℃. The melt-blown nonwoven PP fabrics were fabricated with different basis weights of 15, 30, and 50 g/m^2^.

To form the TPU nanofiber mat on the nonwoven PP fabric, TPU was dissolved in a mixture of DMF and THF (7:3), to obtain a 12 wt% solution. The dissolved solution was electrospun onto the obtained nonwoven PP fabric, without any spacer, through the metal nozzle (25G) connected to a high-voltage generator (NNC-HV30, NanoNC, Seoul, Korea). The applied voltage and feed rate were 18 kV and 0.3 mL/h, respectively. The distance from the metal nozzle to collector was 15 cm. After that, the so-obtained nano- and microfiber nonwoven triboelectric layer was dried at 30 ℃, under vacuum, for 24 h.

### 2.3. Fabrication of N-TENGs

The triboelectric layer consisted of the nonwoven PP fabric (161–475 μm thick) and TPU nanofiber mat (80 μm thick) as electron-acceptor and electron-donor materials, respectively. The two triboelectric layers were directly fabricated by the spinning process without further processing. The porous nonwoven PP/TPU triboelectric layers were cut into 50 mm × 50 mm pieces. One of the pieces was placed between two Ni-coated conductive fabric (45 mm × 65 mm × 0.11 mm) serving as electrodes, and the structure was fixed with conductive adhesives. The so-fabricated TPU/PP N-TENG was examined in the vertical contact mode.

### 2.4. Characterization

The surface and cross-sectional morphologies of the nonwoven PP and TPU nanofiber mat were examined by FE-SEM (SU8010, Hitachi Co., Japan) at an acceleration voltage of 10 kV, after sputter-coating with osmium. The surface and internal topology were also observed by using a 3D digital microscope (RH-2000, Hirox, Tokyo, Japan). In addition, the data for the curved surface diagram were extracted from the cross-sectional topology image. The pore size distribution and porosity of the porous nonwoven PP and TPU nanofiber mat were characterized by a capillary flow porometry (CFP-1500-AEX, PMI Inc., Ithaca, NY, USA), according to ASTM F316-03, and mercury porosimetry (Auto pore IV 9500, Micromeritics, Norcross, GA, USA), respectively. The permeability of the porous nonwoven PP and TPU nanofiber mat was measured by an air permeability tester (FX3300, TEXTEST, Schwerzenbach, Switzerland). The electrical output performance of the porous nonwoven fiber-based TPU/PP N-TENGs was assessed with a digital oscilloscope (DSOX4024A, Keysight, Santa Rosa, CA, USA) and a source meter (B2901A, Keysight, Santa Rosa, CA, USA). The open-circuit voltage (V_oc_) and short-circuit current (I_sc_) of the TENGs with various basis weights of the nonwoven fabric were tested with a constant force of 5 N, at a frequency of 8 Hz. The porous nonwoven TPU/PP50 TENG was examined under different external forces and frequencies. Finally, the N-TENG-based insole was connected to 57 light-emitting diodes (LEDs) and tested under different human motions, such as walking and running.

## 3. Results

**Figure 1:**
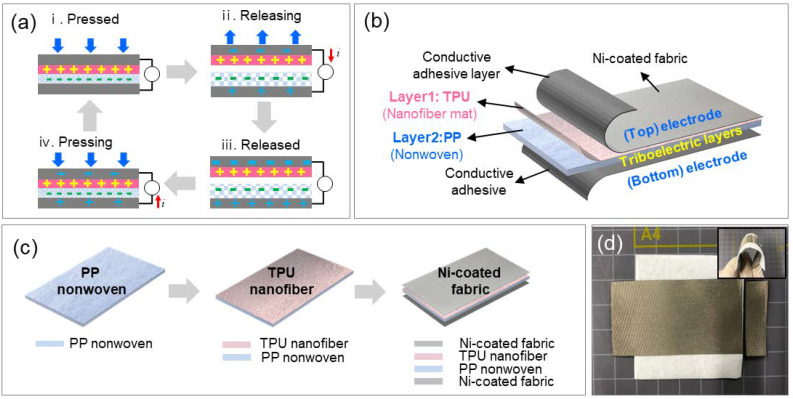
(**a**) Working principle of the TENG in vertical contact-separation mode, (**b**) design of the porous TPU/PP nonwoven fiber-based TENG, (**c**) schematic of the fabrication specimens, and (**d**) the image of a TENG specimen.

Generally, TENG devices are designed to work in four modes, namely the vertical contact-separation mode, in-plane sliding mode, single-electrode mode, and freestanding triboelectric-layer mode. In this work, to apply the wearable generator to footwear, TENGs were designed to operate in the vertical contact mode. Figure 1a shows the general working principle of a TENG in the vertical contact-separation mode. Initially, no charge is generated, because there is no potential difference between the two electrodes [12]. When an external force is applied to the upper fabric layers (Figure 1(ai)), the surfaces of the upper and lower layers come in contact with each other; as a result, electrification take places at the contact area. During contact, the triboelectric layers on both sides generate a charge, which depends on the inherent electrical property of the material. When the external force is released (Figure 1(aii)), the induced positive and negative charges are placed at the bottom and top electrode, respectively. The potential difference across the external circuit can make the electrons to flow promptly. After the complete removal of the external force, the charges are fully equilibrated by the electrostatically induced charging process (Figure 1(aiii)). Under this circumstance, the accumulated charges are not completely dissipated, and they remain in the structure due to the insulator property of polymer materials. After that, when the external force is applied to the two layers, they contact each other and the accumulated induced charge flows back through the external circuit, to balance the electrical potential difference (Figure 1(aiv)). This constitutes one full cycle of the vertical contact-separation mode, and the positive and negative charges are completely offset after returning to the initial state (Figure 1(ai)). Based on this working mechanism, our N-TENG was designed to utilize the nonwoven fiber with a porous structure as the spacer (Figure 1b). We investigated the effect of the porous structure of the nonwoven TPU/PP TENG without spacers on the triboelectric performance for energy harvesting. The spacer-less TENG was constructed from the microfiber-based nonwoven PP fabric and TPU nanofiber mat as the electron acceptor and electron donor of the triboelectric layer, respectively. The fabrication of the nonwoven triboelectric layers is schematized in Figure 1c. First, the porous nonwoven PP fabric was manufactured by using the pilot-scale melt-blowing equipment. Then, the TPU nanofiber was directly fabricated on the surface of the nonwoven PP fabric by electrospinning. Subsequently, this integrated triboelectric layer was placed between two Ni conductive fabrics serving as the top and bottom electrodes. Finally, the structure was fixed with conductive adhesives. Figure 1d shows the photograph of a highly porous nonwoven TPU/PP-based TENG specimen.

**Figure 2:**
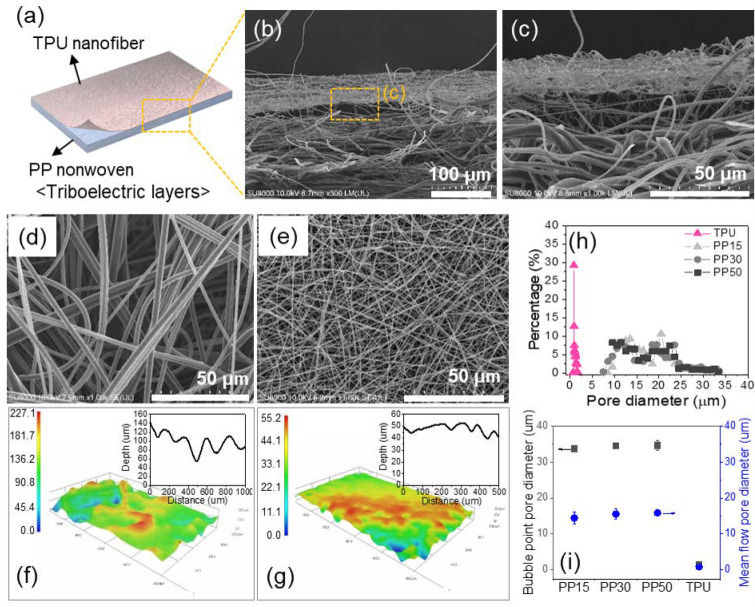
(**a**) Schematic of the highly porous TPU/PP nonwoven triboelectric layers; (**b**,**c**) cross-sectional FE-SEM images of the integrated TPU/PP nonwoven layers; (**d**,**e**) surface morphologies of the porous nonwoven PP and TPU nanofiber mat; (**f**,**g**) 3D surface scanning image and surface roughness graph of the nonwoven PP and TPU nanofiber mat; and (**h**,**i**) pore size distribution and maximum/average pore diameter of both nonwoven fabrics.

Figure 2a shows a schematic of the highly porous TPU/PP nonwoven triboelectric layers developed in this work. The cross-sectional image of the integrated TPU/PP nonwoven layers is shown in Figure 2b,c. Nonwoven PP fabric has a bulky and porous structure with high air volume, whereas the TPU nanofibers are relatively dense and compact along the cross-section. In addition, vacant spaces are observed in the interface layers, which can serve as a spacer between the triboelectric layers. The surface morphologies of the nonwoven PP fabric and TPU nanofiber mat are displayed in Figure 2d,e. The nonwoven PP fabric and TPU nanofiber are randomly distributed, presenting a highly porous structure. The average diameters of the PP nonwoven fiber and TPU nanofiber are 2.6 ± 0.1 μm and 617 ± 43.0 nm, respectively, as shown in Table 1. The diameter of the TPU nanofiber is approximately four times smaller than that of the PP microfiber. Furthermore, the TPU nanofiber mat consists of a more compact and denser structure than that of the nonwoven PP fabric. The thickness of the nonwoven PP50 fabric is approximately ten times higher than that of nonwoven TPU. The 3D topological images of each triboelectric layer are presented in Figure 2f,g. The surface topology of the nonwoven PP fabric and TPU nanofiber mat were observed by the 3D images. The depth analysis of the cross-sectional topology image indicates that the surface of the nonwoven PP fabric is rougher and deeper compared to that of the TPU nanofiber mat. This difference in the surface topology of each constituent material will create empty spaces between the two layers, which can act as a spacer under the absence of an external force. Figure 2h,i present the pore size distributions of the porous TPU nanofiber mat and nonwoven PP fabrics with various basis weights. The pore size distribution of nonwoven PP is relatively broad, and a similar range is observed for specimens with different basis weights. The average and maximum pore sizes (bubble point pore size) of the nonwoven PP are approximately 16.9 and 33.7 μm, respectively. On the other hand, the pore size distribution of the TPU nanofiber mat was significantly narrow, and the average pore size of the TPU mat was significantly smaller than that of its counterpart, nonwoven PP. In addition, the porosity of the nonwoven PP fabric increased slightly with the increasing basis weight, whereas the permeability decreased significantly with increasing basis weight. The TPU nanofiber mat showed a relatively higher porosity by small pore size, although it has a relatively low permeability by the compact structure composed of nanofiber. These results confirm that the micro-sized nonwoven PP, which increased according to the basis weight, has a highly porous bulk structure and abundant air volume. The TPU nanofiber mat is porous but denser and thinner compared to nonwoven PP, and the structural difference between the two nonwoven layers can make the vacant spaces act as a spacer in the interface layer. Furthermore, the contact area of the highly porous nonwoven structure can be increased by applying external force.

**Figure 3:**
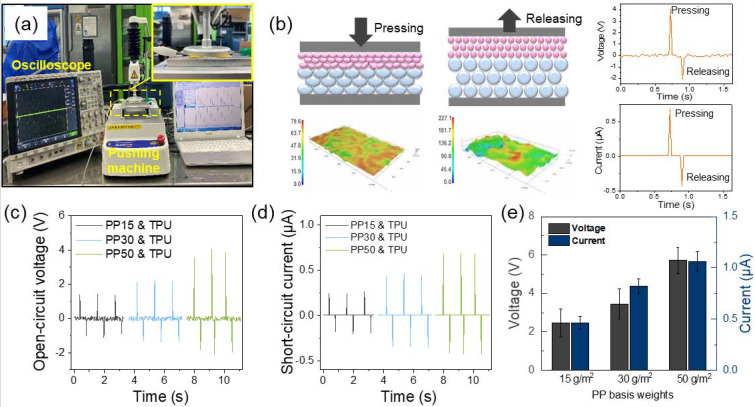
(**a**,**b**) Schematic illustration of the change in the fiber contact area of a compressed and decompressed nonwoven based N-TENG; (**c**) open-circuit voltage and (**d**) short-circuit current of the porous TPU/PP nonwoven TENGs with different basis weights of PP; (**e**) the average output voltage and current of each specimen over 100 cycles.

In order to confirm the effect of the different basis weights of nonwoven PP on the triboelectric performance of the N-TENG, the electrical properties of N-TENGs were investigated by using pressure-applying equipment, as shown in Figure 3a. A schematic of the change in the contact area of the compressed and decompressed nonwoven fiber-based N-TENG is shown in Figure 3b. The nonwoven triboelectric layers have a high surface-area-to-volume ratio and numerous pores with high volumes of air between the fibers. When pressure is applied, the effective surface contact area between the nonwoven mats in the N-TENG increases, because numerous vacant spaces in the triboelectric nonwoven layers contract under the pressure. As the effective contact area reaches its maximum value under the applied force, the output performance will be improved by the generated charges. When the applied pressure is released, the nanofibers and microfibers return to their original position, and the effective contact area is significantly decreased [36]. Figure 3c,d compares the electrical output performance of N-TENGs with different PP basis weights over three cycles. The N-TENG was repetitively pressed at a constant force of 5 N at 8 Hz frequency. As shown in Figure 3c, the output voltages of PP15, PP35, and PP50-based N-TENGs are approximately 2.34 ± 0.13, 3.53 ± 0.12, and 5.79 ± 0.38 V, respectively. This result indicates a linear increase in the output performance, with an increase in the basis weight of nonwoven PP. The maximum voltage reached 5.79 ± 0.38 V for PP50-based N-TENG. Figure 3d shows that the output current of the N-TENG increased gradually with an increase in the PP basis weight. The maximum output current of the N-TENG with TPU/PP50 layers is approximately 1.1 ± 0.01 μA. In order to compare the average of the output voltage and current over 100 cycles, the pressure was applied and released 100 times, using N-TENGS with different PP basis weights. The results confirm that the output voltage and current of N-TENGs increase with an increase in the PP basis weight. Thus, the thickness control of the PP nonwoven layers to hold higher air volume and enable a larger contact area could improve the triboelectric performance of the N-TENG. To investigate further practical application, the best-performing N-TENG with nonwoven TPU/PP50 triboelectric layers was used. 

**Figure 4:**
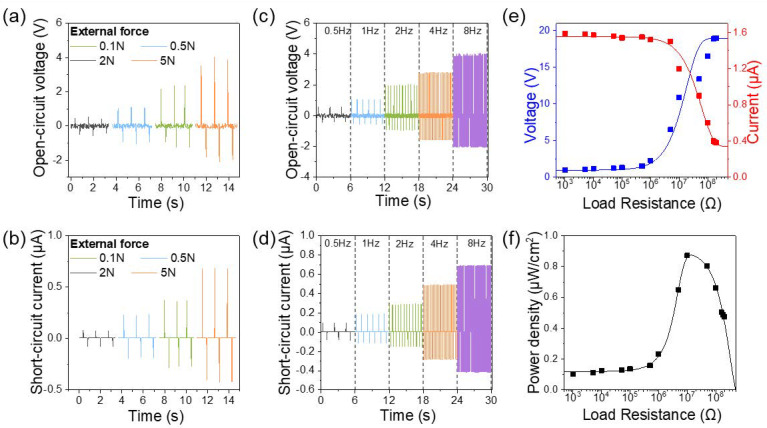
Output performance of the nonwoven TENG. (**a**) V_oc_ and (**b**) I_sc_ of the TENG under different external forces from 0.1 to 5 N. (**c**) V_oc_ and (**d**) I_sc_ of the TENG under different frequencies from 0.5 to 8 Hz. (**e**) Output voltage, current, and (**f**) output power density as functions of external load resistance from 1 kΩ to 100 MΩ, under the applied force of 5 N and frequency of 8 Hz.

Figure 4a,b shows the open-circuit voltage (V_oc_) and short-circuit current (I_sc_) of the TPU/PP50 N-TENGs under different external forces at a fixed frequency at 8 Hz. The results indicate that the V_oc_ and I_sc_ increased from 1 to 5.8 V and from 0.15 to 1.1 μA, respectively, as the external force increased from 0.1 to 5 N. It is clear that the output performance of the N-TENG improves with an increase in the external force. At a lower external force, the fibers of each triboelectric layer make contact over a relatively smaller area in comparison to that achieved under higher external force. As the force increases, a large deformation of N-TENG takes place, leading to increased contact area between the triboelectric layers. This explains why the contact area between the triboelectric layers increases when the applied force is increased, resulting in the improvement of the output performance. Since wearable devices with a nanogenerator are always exposed to physical energy with irregular and varying frequencies, it is essential to evaluate the frequency-dependence of the TENG device [36]. Figure 4c,d presents the output performance of the TPU/PP50 TENG under a fixed external force of 5 N applied at different frequencies. The V_oc_ and I_sc_ of the TENG increase from 1 to 3.63 V and 0.17 to 1.1 μA, respectively, presenting a clearly increasing trend with the frequency in the range of 0.5 to 8 Hz. The fast deformation frequency allows electrons to flow rapidly through the external circuit to reach equilibrium in a short time [1]. The dependence of the power density on the external resistance should be considered for the practical application of N-TENGs. Therefore, the output current and voltage of the N-TENG under external load resistance from 1 kΩ to 200 MΩ were measured at 5 N force and 8 Hz. As the resistance increases, the voltage tends to increase and saturates at approximately 19 V (Figure 4e), although the current decreases. The maximum power density of 0.9 μW/cm^2^ is reached at a load resistance of 10 MΩ (Figure 4f).

**Figure 5:**
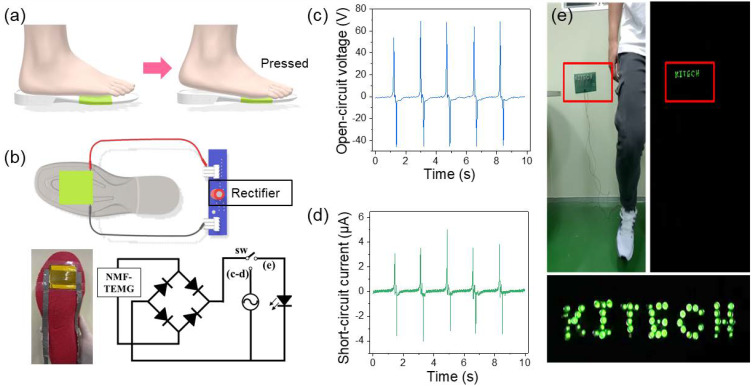
Demonstration of energy generation by human walking. (**a**) Schematics of the working principle and (**b**) circuit configuration for an energy-harvesting insole. (**c**) V_oc_ and (**d**) I_sc_ generated by human walking. (**e**) Photograph of 57 LEDs with stable luminance powered by human walking.

In general, a person usually walks more than 6000 steps per day. In addition, while walking, a person’s load is periodically applied to the foot at an average frequency of 1.8 Hz. Therefore, the walking-based generator is advantageous for generating electrical energy by friction. As shown in Figure 5a, for energy generation by human-walking, N-TENG attached to an insole was inserted into the shoe. In addition, the human-walking-based TENG was used as a power source for a full-wave rectifier circuit, which was used to convert AC signal into pulsating DC signal (see Figure 5b). Furthermore, as shown in Figure 5c,d, the electrical output voltage and current generated by human-walking reached the maximum values of 110.18 ± 6.06 V and 7.28 ± 0.67 µA, respectively. This result indicates that the energy generated by the N-TENG is enough to drive the wearable device. As a direct demonstration, 57 commercial LEDs connected in series in the form of “KITECH” were turned on, using the energy generated by the N-TENG with human-walking, without any charging system, as shown in Figure 5e (see the Supporting Information, Video S1). Therefore, our N-TENG can be applied to a wearable device, as a power source that harvests energy generated by human-walking.

## 4. Conclusions

In summary, we developed highly porous nonwoven TPU/PP-based TENGs, which are flexible and breathable, for wearable devices. We investigated the influence of the basis weight of nonwoven PP on the triboelectric performance of TENGs. Structural characterization revealed that the micro-sized PP nonwoven has a relatively large pore size and high permeability in comparison to that of its counterpart, TPU nanofiber mat, and the porous structure and thickness of nonwoven PP were affected by the basis weight. Owing to the highly porous nonwoven structure with a high air volume, the interface layer between the two counter triboelectric layers were not fully in contact even when they were integrated. Thus, the TENG could harvest energy under different external forces and frequencies. Under walking and running motions, our TPU/PP50 N-TENG provided an output voltage and current of 110.18 ± 6.06 V and 7.28 ± 0.67 µA, respectively. Furthermore, the energy generated by our N-TENG-based insole could be used to directly light up 57 green LEDs sustainably. Thus, we fabricated a facile, breathable, and flexible generator fully based on fibers, which can be applied in wearable devices. In addition, the manufacturing method of the triboelectric layer of our N-TENG is low-cost and amenable to mass production. Therefore, this study can help develop large-area generators that can be applied in self-powered wearable clothes and footwear in the near future.

## Figures and Tables

**Table 1:** Basic properties of the nonwoven PP and TPU nanofiber mat.

Label	Diameter	Thickness (μm)	Air Permeability (L/m^2^/s)	Mean Pore Size	Bubble Point Pore Size	Porosity (%)
PP15	2.5 ± 0.4 μm	180	767.8 ± 51.3	16.1	34.0	77.7 ± 2.9
PP30	2.7 ± 0.3 μm	350	363.2 ± 28.4	17.9	33.8	78.4 ± 1.2
PP50	2.7 ± 0.5 μm	600	270.3 ± 26.4	16.8	33.2	80.1 ± 0.2
TPU	617 ± 43.0 nm	120	6.0 ± 2.1	0.9	1.6	90.6 ± 0.5

## Supplementary Materials

The following are available online at https://www.mdpi.com/2073-4360/12/5/1044/s1, Video S1: The commercial 57 LEDs connected in series in the form of “KITECH” by using the energy generated by the N-TENG with human-walking without any charging system.
