# Supplementary material for: A Highly Porous Nonwoven Thermoplastic Polyurethane/Polypropylene-Based Triboelectric Nanogenerator for Energy Harvesting by Human Walking
Source: Polymers (Basel). 2020 May 2;12(5):1044. doi: 10.3390/polym12051044 (PMC7284548; doi:10.3390/polym12051044)
Supplement: Supplementary file 1 [file polymers-12-01044-s001.pdf]

Supporting information,

S1. The commercial 57 LEDs connected in series in the form of “KITECH” by using the energy generated by the N-TENG with human-walking without any charging system. (Video1)

https://doi.org/10.5281/zenodo.3741388
